# Gynecologic Surgery during the COVID-19 Pandemic: Is Universal Screening Mandatory?

**DOI:** 10.1155/2021/5528334

**Published:** 2021-08-23

**Authors:** Wilasinee Areeruk, Keerati Chiengthong, Somsook Santibenchakul, Shina Oranratanaphan, Tarinee Manchana

**Affiliations:** Department of Obstetrics and Gynecology, Faculty of Medicine, Chulalongkorn University, Bangkok 10330, Thailand

## Abstract

This study is aimed at evaluating the results of the universal preoperative screening for COVID-19 in gynecologic cases operated on during its outbreak in a tertiary care hospital in Bangkok, Thailand. A retrospective descriptive study was done on all patients who underwent elective or emergency gynecologic surgeries during the pandemic period in Thailand (April 15 to June 5, 2020). The COVID-19 screening results by symptom-based screening, risk-based screening, and RT-PCR for COVID-19 were collected from the electronic medical records. Among 129 patients who underwent gynecologic surgeries, none had a positive RT-PCR for COVID-19. Symptom-based screening found no patients with positive symptoms for COVID-19. Risk-based screening found 4 patients (3.1%) who were in contact with suspected or confirmed COVID-19 cases and 4 patients (3.1%) who were healthcare personnel. In conclusion, routine preoperative RT-PCR for COVID-19 may need to be reconsidered among asymptomatic individuals in a low-prevalence country during the well-controlled COVID-19 situation. Larger studies are required to ascertain the benefit of universal preoperative COVID-19 testing.

## 1. Introduction

COVID-19, a disease caused by a novel coronavirus known as severe acute respiratory syndrome coronavirus 2 (SARS-CoV-2), has been identified as the cause of the COVID-19 pandemic in the city of Wuhan, China, since the end of 2019. The outbreak spread quickly in the region and to other parts of the world, causing the World Health Organization (WHO) to respond immediately by providing diagnostic development, issuing guidance on monitoring, treatment, and prevention [1]. Human-to-human transmission through contaminated droplets, aerosols, hands, or surfaces is the main source of transmission. Moreover, transmission by asymptomatic individuals during the incubation period has been reported [2]. Although the infection is less fatal than the previous outbreaks such as SARS (severe acute respiratory syndrome) or MERS (Middle-East respiratory syndrome), it seems to be highly contagious compared to other diseases including influenza [3].

According to the highly contagious nature of COVID-19, surgery has been disrupted worldwide. In performing surgery during the COVID-19 outbreak period, special considerations must be made to prevent transmission to both patients and surgical teams as well as to prevent performing the procedures in asymptomatic patients who are more vulnerable to postoperative morbidity and mortality. Several countries have launched preoperative screening guidelines for COVID-19. Most screening methods are based on symptoms, history of contacting suspicious cases, RT-PCR for COVID-19, serologic test, or preoperative computed tomography (CT) of the chest [4–7]. The preferred diagnostic test for COVID-19 is the RT-PCR of specimens collected by nasopharyngeal or oropharyngeal swabs. Bronchoscopy is not recommended due to the possibility of aerosol generation that poses transmission risk for the patients and healthcare providers [2]. However, the benefits of preoperative screening have not yet been clarified.

Thailand was the second COVID-19 outbreak country with its first case reported on January 12^nd^, 2020. Both imported and local transmission cases increased continuously from less than 10 new cases per day in January, 2020, and reached its peak at 188 new cases per day in late March, 2020 [8, 9]. This prompted the Thai government to announce emergency decrees to respond to the outbreak situation in the same month. During the outbreak, medical equipment, personal protective equipment (PPE), ventilators, and intensive care units were in high demand. Elective surgical cases all over the country were postponed to ensure safety to the patients and surgical teams and also to conserve valuable medical equipment and PPE. Many hospitals launched preoperative screening protocols for COVID-19 and guidelines for cases to be performed.

This study was carried out to assess the results of universal preoperative screening for COVID-19 in gynecologic cases operated on during its outbreak in a tertiary care hospital in Bangkok, Thailand. The incidence of COVID-19 in the country during the study period was 0-53 new cases per day.

## 2. Material and Methods

This retrospective descriptive study was conducted at King Chulalongkorn Memorial Hospital, a tertiary care hospital in Bangkok, Thailand. After approval from the Research Ethics Committee of the Faculty of Medicine, Chulalongkorn University, data were collected from all patients undergoing gynecologic surgeries during April 15 to June 5, 2020.

According to hospital protocol, preoperative screening for COVID-19 was done on all patients who underwent gynecologic surgeries, either elective or emergency, during that period. As the hospital policy was set to lower the operation rate, only those patients whose outcomes might be affected by delayed surgeries, i.e., with confirmed or suspected gynecologic cancer patients, premalignant cases, or benign gynecologic diseases with severe symptoms, were scheduled. COVID-19 screening included symptom-based screening, risk-based screening, and laboratory testing. Symptom-based screening was done by using a questionnaire asking about recent complaint of fever, cough, rhinorrhea, shortness of breath, and sore throat. Risk-based screening was assessed by questionnaire about the possibility of contacting COVID-19 as a result of travel to endemic areas, history of contacting suspicious or confirmed cases, and personal working history in healthcare areas.

Nasopharyngeal swabs were performed on all patients and sent for RT-PCR for COVID-19 using the Cobas® SARS-CoV-2 test (Roche Diagnostics, Indianapolis, IN, USA). Medical residents training in the Obstetrics and Gynecology Department performed nasopharyngeal swabs in a negative pressure chamber. All residents performing the nasopharyngeal swabs were trained by an infectious specialist. If the RT-PCR results were positive, the surgery would be postponed. A “No-visitors policy” was also implemented during that period. Patients were required to wear face masks at all times during their hospital stay.

Data analysis was done using the SPSS software package version 22.0 (SPSS Inc., Chicago, USA). Continuous data were presented by mean (standard deviation (SD)) or median (interquartile range (IQR)), while categorical data were presented by number and percentage.

## 3. Results

During the 52-day period (April 15 to June 5, 2020), there were 122 patients scheduled for elective gynecologic surgeries and 9 patients who underwent emergency gynecologic surgeries. One gynecologic cancer patient was canceled due to neutropenia, and another had thrombocytopenia. For the remaining 129 patients (patients with conditions that surgeries could not be postponed), baseline characteristics are shown in Table 1. Most patients were Thai (98.4%), with a median age of 48 years. There were 33 patients with presumed or underlying cancers including 24 patients that were scheduled for gynecologic cancer surgeries, 4 breast cancer patients, 2 lymphoma patients, and 1 gastric cancer patient, and the other 2 patients were presumed to be endometrial cancer patients who underwent fractional curettage.

Symptom-based screening found no patients with positive symptoms for COVID-19. Risk-based screening, assessed by a questionnaire, found 4 patients (3.1%) who came in contact with suspected or confirmed COVID-19 cases and another 4 patients (3.1%) that were healthcare personnel who were considered high-risk contact for COVID-19 cases. No patients traveled from endemic countries (Table 2).

Nasopharyngeal swab RT-PCR for COVID-19 was done in all 129 cases. The results were all negative. Hospital protocol dictated that in emergency cases where the results could not be reported prior to the operation, full PPEs were used by all medical staff in the operating room.

The operative characteristics are shown in Table 3. There were 120 elective surgical cases (93.0%) and 9 emergency surgical cases (7.0%). Major operations were done in 78 patients (60.5%). Minor operations including curettage, cervical excisional procedure, incisional, and drainage or marsupialization were done in 51 patients (39.5%). There were 24 gynecologic cancer patients who were operated on during that period (18.6%) and 10 cases that underwent laparoscopic surgeries during that period (7.7%).

Postoperative complications are shown in Table 3. Eight patients (6.2%) required ICU admission. Small numbers of other complications were found including bowel injury (0.8%), bladder injury (0.8%), and wound infection (0.8%). There was one case with postoperative respiratory complication. The patient had ovarian mucinous cystadenoma and underwent total abdominal hysterectomy with bilateral salpingo-oophorectomy with surgical staging due to preoperative suspicion of ovarian cancer. She developed fever during the postoperative period. The postoperative chest radiography showed patchy infiltration at the left lower lung zone compared with her preoperative normal chest radiography. She had no respiratory symptoms, and the chest radiography improved significantly in the next 48 hours. Aspiration pneumonitis was diagnosed.

All patients were followed up postoperatively at 2 weeks as per hospital protocol for pathological reports and routine postoperative checkup. None of the patients had postoperative complications or developed COVID-19 during this period.

## 4. Discussion

This study was done to assess the universal preoperative screening of COVID-19 prior to gynecologic surgery in Thailand. The RT-PCR results were all negative in our study. As previously mentioned, this led us to question whether or not preoperative RT-PCR for COVID-19 screening is mandatory in our country. To date, few studies have addressed the benefits of preoperative screening for COVID-19. The Centers for Disease Control and Prevention (CDC) recommends the use of nasopharyngeal swabs for the assessment of COVID-19 infection in both symptomatic and asymptomatic individuals and also states that COVID-19 testing before a procedure might be beneficial for public health reasons [10]. The CDC preoperative screening guidance is recommended by the American Society of Anesthesiologists [11]. The Royal College of Obstetricians and Gynaecologists has also recommended that patients scheduled for elective surgery should undergo COVID-19 screening with oropharyngeal or nasal swabs [12].

Previous data in the US reported 12% positive preoperative screening tests prior to otolaryngologic surgery [13]. In another large multicenter study in the States, there were 0.58% positive preoperative screening tests among 1198 patients undergoing orthopedic surgery [14]. Another preoperative screening study in Malaysia reported no positive screening tests in low-risk asymptomatic patients [15]. The study of preoperative CT chest in the UK reported 7% of patients with positive COVID-19 related lung change, but only 1.6% had confirmed COVID-19 by RT-PCR [16]. There are limited data on preoperative screening prior to gynecologic surgery. A study in Spain reported 4 out of 64 patients (6.2%) with positive RT-PCR for COVID-19 prior to gynecologic cancer surgeries [17]. The varying results could be explained by the difference in prevalence of COVID-19 among each region and the different screening techniques (Table 4).

Thailand as the second COVID-19 outbreak country, the number of cumulative COVID-19 cases increased at a slow rate from January to mid-March, 2020. The increasing incidence reached its peak at over one hundred cases per day in late March to early April, then returned to the previously slow rate afterwards [18]. We suspected this well-controlled situation to be due to the nationwide lockdown from March, 2020. The preoperative screening period in our study was when the situation in our country was well-controlled with a mean number of new cases at 10.24 cases per day [19]. This might explain why there are no positive RT-PCR cases in our study. As of October, 2020, Thailand's Global COVID-19 Index (GCI) rank was 3^rd^ worldwide with a recovery index of 82.37 out of 100, which confirms the effective handling and recovery from the COVID-19 crisis. This GCI rank also confirmed the well-controlled situation in the country [20].

Although nasopharyngeal swab RT-PCR is the preferred diagnostic test for COVID-19, the sensitivity in asymptomatic patients remains unclear [21]. False-negative results have been reported ranging from 11 to 40% [22]. It must be raised that this can initiate a false sense of security during an operation. We believe that droplet precaution should still be used in suspicious cases regardless of RT-PCR result.

The limitation of the study is that this is a single-center study with limited numbers of patients. This limitation is due to (1) the short period of COVID-19 outbreak in our country, (2) selection of only those patients with conditions that surgeries could not be postponed, and (3) only patients without COVID-19 related symptoms that were scheduled for the operations. As a result, no positive RT-PCR screening was found in our population. Patient characteristics in this study were similar to a previous study conducted in 2014 in our hospital; therefore, it would represent the general gynecologic patient population at the hospital despite the small sample size limitation [23].

We suppose our data to be used as a reference for developing preoperative screening guidelines for COVID-19 in the future. It can be of value prior to the second wave of the pandemic in similar GCI countries. However, a further study with larger sample size should be done to provide more data on this topic. Our recommendations should be used with caution when applying to high prevalence countries.

In conclusion, as the incidence of COVID-19 in Thailand has decreased, the number of gynecologic surgeries performed has increased with a return to full-scale surgery since June, 2020. Based on our data, routine preoperative RT-PCR for COVID-19 may need to be reconsidered in low-prevalence countries with well-controlled situations. However, symptom-based and risk-based screening protocols are still recommended in all patients prior to performing the surgery. Larger studies are required to ascertain the benefit of routine preoperative COVID-19 testing.

## Figures and Tables

**Table 1:** Patient characteristics.

	*N* = 129
Age (years), median (IQR)	48 (38.5-59.5)
Ethnicity, *n* (%)	
Thai	127 (98.4%)
Others^∗^	2 (1.6%)
BMI (kg/m^2^), mean (SD)	24.00 (4.64)
<18.5 kg/m^2^, *n* (%)	10 (7.8%)
18.5–22.9 kg/m^2^, *n* (%)	55 (42.6%)
23.0–24.9 kg/m^2^, *n* (%)	22 (17.1%)
≥25.0 kg/m^2^, *n* (%)	42 (32.6%)
Hospital stay (days), median (IQR)	3 (0.5-4)
Menopausal status, *n* (%)	
Premenopause	79 (61.2%)
Postmenopause	50 (38.8%)
Underlying disease, *n* (%)	
Diabetes mellitus	15 (11.6%)
Hypertension	24 (18.6%)
Respiratory disease	0 (0%)
Cardiovascular disease	4 (3.1%)
Cancer	33 (25.6%)

^∗^South Africa and Ukraine.

**Table 2:** COVID-19 screening results.

	*N* = 129
Risk-based screening, *n* (%)	
Recent travelling from COVID-19 endemic regions	0 (0%)
Contact with suspected or confirmed COVID-19 patients	4 (3.1%)
Healthcare personnel	4 (3.1%)
Symptom-based screening, *n* (%)	
Fever	0 (0%)
Cough	0 (0%)
Rhinorrhea	0 (0%)
Shortness of breath	0 (0%)
Sore throat	0 (0%)
Laboratory, *n* (%)	
Positive RT-PCR for COVID-19	0 (0%)

**Table 3:** Operative characteristics.

	*N* = 129
Type of surgery, *n* (%)	
Elective	120 (93.0%)
Emergency	9 (7.0%)
Operation, *n* (%)	
Major operation	78 (60.5%)
Minor operation	51 (39.5%)
Diagnosis, *n* (%)	
Benign	105 (81.4%)
Malignancy	24 (18.6%)
Operative route	
Laparotomy	63 (48.8%)
Laparoscopy	10 (7.8%)
Hysteroscopy	6 (4.7%)
Vulva/vaginal operation	50 (38.8%)
Operative complication, *n* (%)	
ICU admission	8 (6.2%)
Blood loss > 1000 ml	8 (6.2%)
Bowel injury	1 (0.8%)
Bladder injury	1 (0.8%)
Wound infection	1 (0.8%)
Pneumonia (aspiration)	1 (0.8%)

**Table 4:** Data on preoperative screening for COVID-19.

Study	Country	Surgery	No. screened	% positive	Screen method	Screen period	Local incidence (new cases/d)
Urban MJ, et al.	Illinois, USA	Otolaryngologic surgery	25	12%	Nucleic acid amplification test	Mar 23-Apr 17, 2020	238-1841
Blumberg TJ, et al.	USA	Pediatric orthopedic surgery	1198	0.58%	RT-PCR	Mar-Jun, 2020	0-7947
	Philadelphia		373	1.07%			0-1023
	Seattle		299	0.33%			8-231
	Texas		526	0.38%			0-7947
Sii CKS, et al.	Malaysia	Pediatric surgery	66	0%	RT-PCR	Mar 18-May 31, 2020	0-235
Chetan MR, et al.	UK	All surgery	439	1.6%	CT chest, confirmed by RT-PCR	Mar 25-Apr 27, 2020	2693-5494
Santiago JD, et al.	Spain	Gynecologic cancer surgery	64	6.2%	RT-PCR	Mar-May, 2020	36-9222
Areeruk W, et al. (this study)	Thailand	Gynecologic surgery	129	0%	RT-PCR	Apr 15-Jun 5, 2020	0-53

## Data Availability

The data used to support the findings in this study are available from the corresponding author upon request.
